# Perturbative countersurveillance metaoptics with compound nanosieves

**DOI:** 10.1038/s41377-019-0212-4

**Published:** 2019-11-15

**Authors:** Jiancai Xue, Zhang-Kai Zhou, Limin Lin, Chao Guo, Shang Sun, Dangyuan Lei, Cheng-Wei Qiu, Xue-Hua Wang

**Affiliations:** 1 State Key Laboratory of Optoelectronic Materials and Technologies, School of Physics, Sun Yat-sen University, 510275 Guangzhou, China; 2 Department of Electrical and Computer Engineering, National University of Singapore, 4 Engineering Drive 3, Singapore, 117583 Singapore; 3 Department of Materials Science and Engineering, City University of Hong Kong, 83 Tat Chee Avenue, Kowloon, Hong Kong

**Keywords:** Optical materials and structures, Nanophotonics and plasmonics, Applied optics

## Abstract

The progress of metaoptics relies on identifying photonic materials and geometries, the combination of which represents a promising approach to complex and desired optical functionalities. Material candidate options are primarily limited by natural availability. Thus, the search for meta-atom geometries, by either forward or inverse means, plays a pivotal role in achieving more sophisticated phenomena. Past efforts mainly focused on building the geometric library of individual meta-atoms and synthesizing various ones into a design. However, those efforts neglected the powerfulness of perturbative metaoptics due to the perception that perturbations are usually regarded as adverse and in need of being suppressed. Here, we report a perturbation-induced countersurveillance strategy using compound nanosieves mediated by structural and thermal perturbations. Private information can be almost perfectly concealed and camouflaged by the induced thermal-spectral drifts, enabling information storage and exchange in a covert way. This perturbative metaoptics can self-indicate whether the hidden information has been attacked during delivery. Our results establish a perturbative paradigm of securing a safer world of information and internet of things.

## Perturbative metasurface for safe hiding of secret messages

An optical perturbative metasurface incorporating differently shaped nano-sized holes can conceal information and alert the receiver if an unwelcome attempt has been made to uncover it. The perturbative metasurface was proposed and demonstrated by researchers from China and Singapore. The team took advantage of tiny ‘perturbations’ that happen within the metasurface, and thus to its optical properties for nano-steganography, turning perturbations from being useless in normal perceptions to be of significant importance. Based on judicious perturbations, a secret message can be almost perfectly concealed within a visible camouflage image produced by the metasurface and only revealed when the right amount of heat is applied, followed by a matched light filter. The receiver is alerted to an attempted hack, as heat application causes irreversible changes of the camouflage image without revealing the hidden message.

## Introduction

Metaoptics has enabled tremendous fundamental discoveries and novel applications^1–5^, which not only further our understanding of the world but also enrich our daily life with wonders. For example, with the development of metamaterials and metasurfaces, fascinating effects and profound applications are constantly revealed^6–11^, such as electromagnetically induced transparency^6^, a negative refractive index^7^, an ultrathin metalens^9,10^, and a functional thermal meta-device^12^.

These progresses in the field of metaoptics result from designing functional geometries and developing new material components or combining them together^13–17^ to realize complex optical functionalities. Since the photonic material library is primarily limited by natural availability, searching for meta-atom geometries plays a crucial role in realizing more sophisticated functionalities in metaoptics. A general way in this research is to build a library of individual geometric meta-atoms and then to integrate a variety of them into a design to achieve the desired phenomena. In this strategy, perturbations (i.e., the small deviation of structural parameters) are usually regarded as adverse to the designed functionalities and in need of being suppressed, especially in systems with high quality factors^18–20^ and structures with sizes down to the nanoscale^21–26^. However, this factor results in neglecting the powerfulness of perturbations as a positive component in metaoptics, removing the possibility to open up the research world of perturbative metaoptics, which may be rich in interesting phenomena and promising applications. In fact, perturbations have recently been used to theoretically solve the problem of reverse design in elastic metamaterials^27^, which indicates the potential of supportive perturbations in material engineering under the appropriate design.

Here, we explore the use of perturbations in expanding the functions of metaoptics by proposing a perturbation-induced countersurveillance strategy with compound nanosieves. While considerable structural changes in the compound nanosieves are used for camouflage information expression, controllable structural perturbations without a visible influence on the expressed information are used for information hiding. Then, with the designed structural and thermal perturbations, the function of countersurveillance supporting the self-indication of eavesdropping is developed, which is of great importance for information science but has not yet been achieved in metaoptics-based and most other information security techniques. In addition, private information can be almost perfectly concealed and camouflaged by this perturbative countersurveillance strategy, giving rise to almost perfect information hiding for covert information storage and exchange. Our findings not only demonstrate perturbation as a functional dimension in the design of metaoptical systems but also propose a perturbative security strategy for information and the internet of things.

## Results

### The origin of perturbative metaoptics

Metamaterials and metasurfaces have recently enabled a number of novel advanced information security approaches with advantages of high capacity and security^28–34^, opening up a new avenue for applications of metaoptics. However, a common problem in these implementations is that the carriers of the contained information are suspicious and thus easy to draw the attention of others, which may attract eavesdroppers and lead to the failure of information security. To address this problem, in the following, we explore perturbations in metaoptics, which help in realizing additional new functions in an original system. Specifically, they can be used to implement covert information storage and transmission without the awareness of unauthorized third parties by concealing private information under innocuous cover information.

**Fig. 1:**
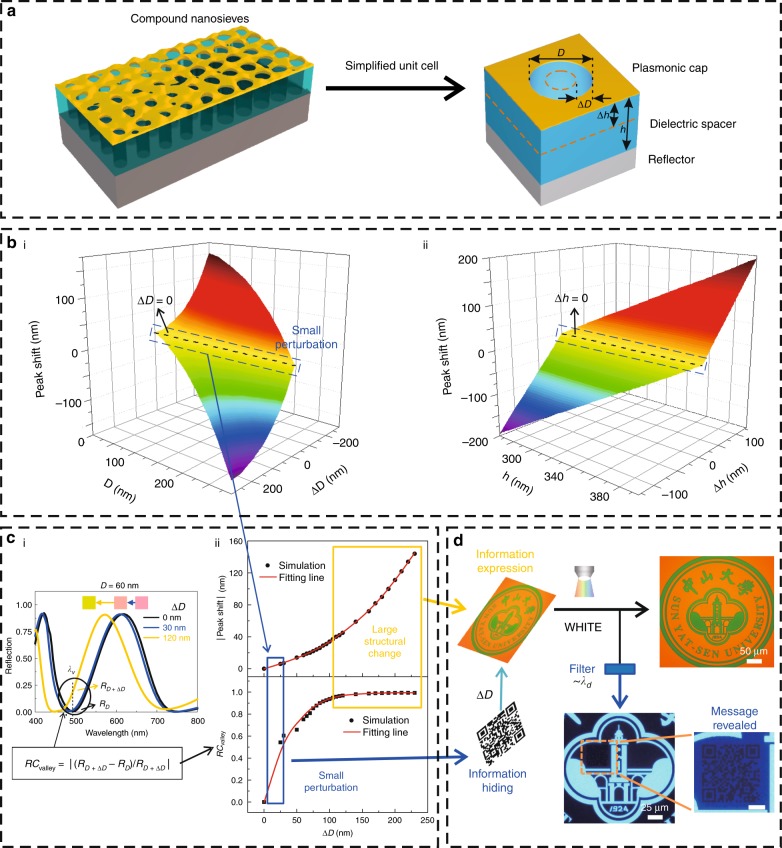
Spectral properties of the compound nanosieves regarding perturbations and their application in high-capacity information hiding. **a** Schematic diagram of the compound nanosieves with a unit cell consisting of a plasmonic cap, a dielectric spacer and a reflector. **b** Peak shifts of reflection spectra against structural changes. i The relationship between peak shifts and Δ*D* at different original *D* values. ii The relationship between peak shifts and Δ*h* at different original *h* values. The dashed black lines represent the control peaks, where Δ*D* *=* 0 in i and Δ*h* *=* 0 in ii with zero peak shift. **c** Spectral difference between the small perturbation region and the large structural change region. i The simulated reflection spectra of the compound nanosieves with *D* = 60 nm and Δ*D* = 0 nm (black curve), 30 nm (red curve) and 120 nm (blue curve). The inserted color blocks are the corresponding colors. ii The relationships between both the peak shifts and *RC*_valley_ (reflection contrast at the original valley wavelength *λ*_*v*_) and Δ*D* with a given diameter *D* = 60 nm. The small perturbation region and the large structural change region are marked by the blue square and the yellow square, respectively. **d** An implementation of information hiding based on the perturbations in the compound nanosieves. A QR code (hidden information) was concealed in a badge of Sun Yat-sen University (expressed information as camouflage) by a small perturbation (Δ*D* = 10 nm), which was invisible under white light (upper-right image). The concealed message was revealed by applying a narrowband filter with designed wavelengths ~*λ*_*d*_ = 480 nm (the images in the lower right). The scale bar in the magnified image of the QR code is 10 μm

Figure 1a shows the compound nanosieves, constructed by unit cells consisting of a plasmonic cap, a dielectric spacer, a nanohole inside them and a reflector at the bottom (Fig. 1a). The plasmonic cap is a rough metallic layer, working as a broadband absorber that helps to generate vivid reflective colors in a wide color range (detailed discussions of these compound nanosieves, as well as the rough plasmonic cap can be found in Figs. S1, S2). In the compound nanosieves, the key parameters are the height (*h*) of the dielectric spacer and the diameter (*D*) of the nanoholes, which directly modulate the reflective peak wavelengths (Fig. S2). When *D* or *h* changes by a large amount (large Δ*D* and large Δ*h* shown in Fig. 1b), the reflective peak wavelengths of the compound nanosieves will undergo a large shift (the regions far away from the yellow regions in Fig. 1b). In contrast, small perturbations of *D* or *h* caused only slight wavelength offsets (regions in the dashed blue squares around the lines of Δ*D* *=* 0 nm in Fig. 1b, i and Δ*h* *=* 0 nm in Fig. 1b, ii). Therefore, the large change regions can be used for information expression, while the small perturbation regions may work for information hiding.

To be specific, for *D* = 60 nm and Δ*D* = 0, 30, 120 nm (the black, blue and yellow spots marked in Fig. 1c, i, respectively), small Δ*D* (30 nm, blue curve) resulted in only a slight spectral shift and little change in the reflective color, while a distinct spectral shift and obvious color change appeared in the case of large Δ*D* (120 nm, yellow curve). According to these facts, a sufficiently modest perturbation of the structure parameter may produce a very small offset in the reflection spectra, resulting in a color difference so tiny that it cannot be discriminated by human eyes, which could be used to hide information.

On the other hand, the reflectivity at the reflective valley could have a large relative increase because of the small valley reflection. To investigate this property more clearly, we focused on the reflection contrast (*RC*) at the original valley wavelength, which was calculated by *RC*_valley_ = |(*R*_*D+*Δ*D*_−*R*_*D*_)/*R*_*D+*Δ*D*_|, where *R*_*D*_ and *R*_*D+*Δ*D*_ are the reflectivity at the valley wavelengths corresponding to Δ*D* = 0 nm and Δ*D* ≠ 0, respectively. As shown in Fig. 1c, ii, *RC*_valley_ increased rapidly as Δ*D* increased in the structural perturbation region (marked by the blue square), while the corresponding peak shifts increased only slightly and remained small. This outcome means that the slight spectral offset caused by a structural perturbation may create sufficient *RC*_valley_, giving rise to visible contrast under the illumination of monochromatic light with the valley wavelength corresponding to Δ*D* = 0 nm. In contrast, in the large structural change region (marked by the yellow square), the reflective peak shifts were quite large and increased rapidly as Δ*D* increased, which is beneficial for constructing cover information in the form of colorful images. The case of Δ*h* is similar to that of Δ*D* and was discussed in Fig. S3. A detailed theoretical discussion on information hiding based on this perturbation-based metaoptics can be found in Fig. S4.

As an experimental demonstration of camouflaged information hiding based on this perturbative metaoptics, a private message (a quick response, QR, code) was transferred into a spatial distribution of structural perturbations in the cover information (a badge of Sun Yat-sen University), making the embedded information invisible under ambient circumstances (Fig. 1d). A modest structural perturbation (Δ*D* = 10 nm) between the QR code pattern (*D* = 65 nm) and neighboring areas (*D* = 75 nm) produced only a small offset in the reflection spectra (see Fig. S5) and resulted in invisibility of the QR code (upper-right image in Fig. 1d), concealing the existence of private information. In the revealing process, the hidden QR code could be revealed clearly (the lower-right images in Fig. 1d) by applying a near-monochromatic light filtered from the microscope-inserted lamp using a narrowband filter centered at the designed wavelength, *λ*_*d*_ = 480 nm (near the reflective valley wavelength shown in Fig. S5). On the other hand, if the wavelength of the light source moves away from the reflective valley wavelength, it will lose the ability to reveal information (see Fig. S6). The *D* adopted in the compound nanosieves is an important parameter in our strategy, and we found that a smaller hole results in better performance. The reason is that the optical properties of the compound nanosieves with smaller diameters are less sensitive to the displacement of diameters (Fig. S7). As a consequence, the concealed information with a fixed ∆*D* and a smaller *D* is more difficult to detect by optical methods when compared with the cases of larger *D*, which is more desirable in information hiding. However, from the experimental perspective, smaller nanoholes correspond to a larger fabrication challenge, and the minimal diameter of the nanohole is also dependent on the equipment limitation. Taking these facts into account, we found *D* = 65 nm to be a suitable value as one practical option.

### Scheme of perturbative countersurveillance strategy

The perturbations in metaoptics permit two advantages for their application in information hiding. One advantage is that the private information embedded in the perturbative compound nanosieves can be transferred by tremendous channels with high secrecy. Since color patterns (the form of camouflage) are ubiquitous in daily life and information can be concealed within a tiny size, such as the QR code in Fig. 1, with a total size (only 34.8 μm) smaller than a pixel on a smart phone screen, the hidden information can be embedded into daily objects or attached to arbitrary items without causing suspicion. The other advantage is that we can obtain resolutions reaching the optical limit, leading to an ultrahigh information capacity of 100,000 d.p.i. (see Fig. S8 for a more detailed discussion). Despite these advantages, an important problem in the elementary form of camouflaged information hiding demonstrated above is that the receiver cannot ensure whether the private information is leaked or not because the hidden information can be read numerous times in the right manner but without a trace. In short, the function of countersurveillance cannot be achieved by this elementary form of perturbative metaoptics (note that this function is still unable to occur in the majority of the existing information security methods).

**Fig. 2:**
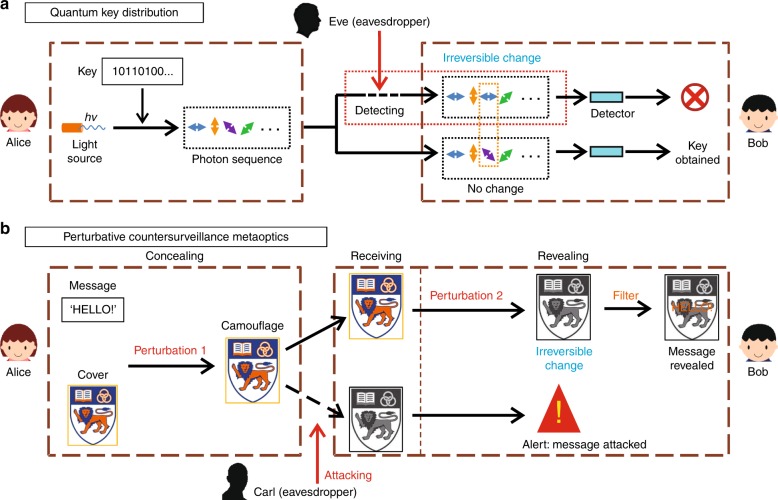
Scheme of the perturbation-induced countersurveillance strategy inspired by quantum key distribution. **a** A simplified scheme of quantum key distribution. Generally, in the quantum key distribution, the message sender, Alice, distributes a key pad by coding it into polarizations of a single photon sequence. After the message receiver, Bob, receives the key pad, he uses it to communicate (the lower case of Bob). However, if there is an eavesdropper, Eve, the behavior of eavesdropping in the channel causes irreversible changes to the key pad. Then, Bob can detect these changes, and he abandons this key pad (the upper case of Bob). This characteristic of quantum key distribution promises unconditional security in information communication. **b** The scheme of the perturbation-induced countersurveillance strategy. At first, Alice embeds a private message (‘HELLO’) in a cover camouflage by introducing invisible perturbation 1. After Bob receives the cover, perturbation 2 accompanied by irreversible changes to the camouflage should be introduced before a filter with the designed wavelengths can be used to reveal the concealed message (the upper case of Bob). This irreversible change works as an indicator of countersurveillance, i.e., if this indicator already appears in the received cover before revealing, Bob will know that an eavesdropper, Carl, has attacked the private information (the lower case of Bob)

Quantum key distribution^35–37^ has gained a remarkable reputation due to unparalleled performances on countersurveillance and its absolute security. In the process of quantum key distribution, the keys can be obtained only by measuring them in a certain way. If a third party (Eve) tries to eavesdrop on the keys, his or her measurements will introduce detectable anomalies, which would be noticed by the receiver (Fig. 2a). Inspired by the quantum key distribution, the function of countersurveillance can be achieved if an irreversible change can occur during the information revealing. Based on this idea, a scheme of perturbative countersurveillance metaoptics was constructed, as shown in Fig. 2b. In the concealing process, an optically invisible perturbation (perturbation 1) is used to conceal private messages in cover camouflage by Alice, the message sender. After the cover carrier is received, a detectable feature of the hidden information does not emerge until perturbation 2 is added by Bob, the message receiver. In this case, the filter with the designed wavelengths can be used only to reveal the concealed message after perturbation 2 is implemented, and the employment of perturbation 2 can lead to irreversible change to the cover image without damaging the hidden information. Therefore, the receiver can judge whether the hidden information has been attacked or not by the appearance of the cover camouflage (Fig. 2b). In the following, we demonstrate the implementation of this scheme in an experiment.

### Perturbation-induced countersurveillance with compound nanosieves

**Fig. 3:**
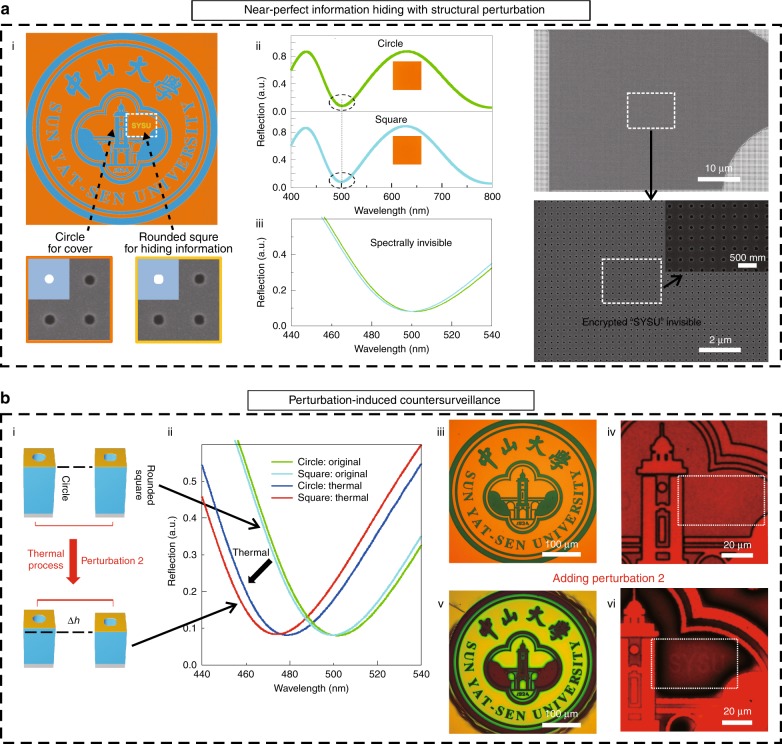
An experimental implementation of the perturbative countersurveillance metaoptics with almost perfect information hiding. **a** Near-perfect information hiding with structural perturbation. i A scheme of the camouflaged information hiding, where the small perturbation between circular holes and rounded square holes is adopted as perturbation 1 to conceal private information. The two inserted SEM images show the corresponding experimental samples with 2 × 2 periods (600 × 600 nm^2^). ii The measured reflection spectra correspond to the compound nanosieves based on circular holes (*D* = 65 nm) and rounded square holes (width *L* = 61 nm), with their corresponding colors inserted. iii The magnified reflection spectra in the areas marked by dashed black circles in ii. iv The morphologies of the area containing concealed messages observed by scanning electron microscopy at different magnifications. **b** Perturbation-induced countersurveillance with compound nanosieves. i A thermal process is able to introduce an *h* perturbation as perturbation 2 because of the different SV ratios caused by perturbation 1. ii The measured reflection spectra of the circle-based and rounded-square-based compound nanosieves before (green and cyan curves, respectively) and after (blue and red curves, respectively) the thermal process, showing the enlarged spectral offset after the introduction of perturbation 2. iii–iv The microscope images of the cover camouflage under the illumination of white light (iii) and narrowband light (iv), showing no trace of the concealed information. v An image of the cover camouflage after adding perturbation 2, showing irreversible changes in colors and color boundaries, which are the visible indicators of countersurveillance. vi An image showing the embedded message revealed by filtered narrowband light with wavelengths centered at *λ*_*d*_ = 690 nm

To construct an optically invisible perturbation working as perturbation 1 in the concealing process, we delicately designed the shapes of the holes in the compound nanosieves and applied circular holes and rounded square holes to form a cover image and embed a hidden message, respectively, as shown in Fig. 3a, i. In the compound nanosieves, the reflection spectra can be tuned to overlap well with each other by achieving the same porosity, even though the shapes of the holes may differ (Fig. S9). Accordingly, by adjusting the sizes of the used circular holes and rounded square holes, the reflection spectra of these two compound nanosieves were tuned to be nearly the same as each other (Fig. 3a, ii, iii). Therefore, the small structural perturbation was spectrally invisible, providing the perturbative compound nanosieves with the ability to make the embedded message optically disappear into the background of the cover camouflage. In addition, since the sizes of the holes were selected to be quite small (~60 nm) and the perturbations were slight, the concealed message was also difficult to distinguish even though the morphology of the compound nanosieves was mapped at the nanoscale, making it morphologically invisible (Fig. 3a, iv and Fig. S10). As a consequence, the perturbative compound nanosieves were provided with the capability of near-perfect information hiding to keep the embedded message invisible both in optical response and in morphology. It is worth noting that this capability has long been highly desired in information security but has yet to be realized in previous works based on photonic methods.

To introduce the function of countersurveillance and to generate detectable features of the concealed information in the revealing process, perturbation 2 based on the thermal effect of the compound nanosieves was then developed to realize perturbation-induced countersurveillance of the camouflaged information hiding (Fig. 3b). Specifically, the resist working as the spacer in the compound nanosieves shrank when baked because of the evaporation of the residue of the solvent, and the compound nanosieves with rounded square holes shrank more than those with circular holes during this thermal process since they had relatively larger exposed surface-to-volume (SV) ratios (Fig. 3b, i). A more detailed discussion of this thermal effect can be found in Fig. S11. This small *h* perturbation induced by the thermal process consequently caused detectable features and a slight spectral offset in the reflection spectra (Fig. 3b, ii).

As an experimental demonstration of the function of perturbation-induced countersurveillance, we utilized perturbation 1 to conceal private information, with rounded square holes for an embedded message of ‘SYSU’ and circular holes for a cover camouflage (Fig. 3b, iii). As shown in Fig. 3b, iii, the embedded ‘SYSU’ was well hidden in the cover badge under normal light illumination and remained invisible when filters were applied because of its good overlapping of the reflection spectra with the camouflage background (Fig. 3b, iv and Fig. S12).

To detect the concealed information, a thermal process should be applied first to introduce perturbation 2. After the implementation of the thermal process, baking at 180 °C for 1 min in this case, the concealed message was still out of sight, although the cover camouflage had a distinct transition of its appearance (Fig. 3b, v and Fig. S13). In addition, the irreversible evaporation of the solvent residue made these structural changes irreversible, bringing about visible indicators including color transitions of the cover camouflage and vague boundaries between different colors. These indicators can work as an inbuilt security alarm for countersurveillance, and serving as a warning for the receiver that alerts the receiver of an attack if these indicators show up when the sample is received. With the added perturbation 2, the embedded ‘SYSU’ was clearly revealed by the application of filtered light with designed wavelengths (Fig. 3b, vi), completing the information revealing process. In addition, the hidden information was also morphologically invisible after the emergence of perturbation 2 (Fig. S14), stemming from the fact that the rounded square holes with a shape similar to that of the circular holes was deliberately chosen (the inserted SEM images in Fig. 3a, i).

### Robust perturbative information hiding

**Fig. 4:**
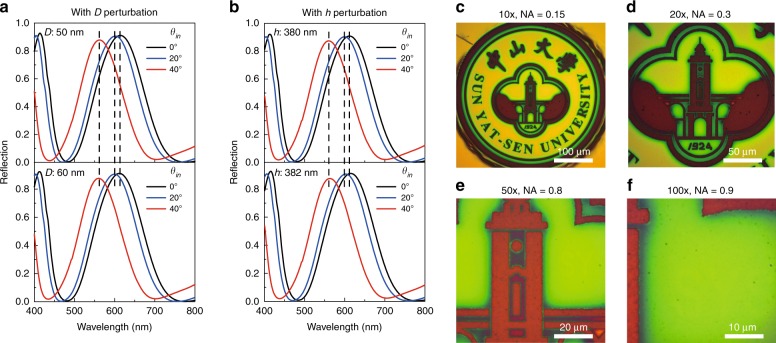
Angle-independent information hiding in the perturbative countersurveillance metaoptics. **a**, **b** Simulated angle-dependent reflection spectra of the compound nanosieves with *D* perturbation (**a**) or *h* perturbation (**b**), showing no enlarged spectral drifts with the increase in incident angles of the observing light. The spacer thicknesses of the compound nanosieves in **a** are 380 nm, and the hole diameters are 50 nm (upper case) and 60 nm (lower case). The hole diameters of the compound nanosieves in **b** are 60 nm, and the spacer thicknesses are 380 nm (upper case) and 382 nm (lower case). **c–f** Images of the cover badge (after the introduction of perturbation 2) in Fig. 3b under white light illumination, observed with objectives of ×10 (NA = 0.3) (**c**), ×20 (NA = 0.45) (**d**), ×50 (NA = 0.8) (**e**), and ×100 (NA = 0.9) (**f**), which correspond to the maximum illuminating angles 17.5°, 26.7°, 53.1°, and 64.2°, respectively. As the objectives changed from ×10 to ×100, the colors of the background changed from yellow to green because of the angle dependence of the compound nanosieves, but the concealed message (‘SYSU’) steadily remained invisible, verifying the robust information hiding ability of the perturbative countersurveillance metaoptics

In a material system with a designed feature size down to the nanoscale, its optical response often depends on the incident angle^1–5^. Therefore, to test the robustness of the camouflaged information hiding with the perturbative countersurveillance metaoptics, we examined the optical angle dependency and found that the angle-dependent responses of the compound nanosieves have little influence on the invisibility of the concealed information, as described in the following. Theoretically, the compound nanosieves with and without perturbations, which can correspond to the concealed message and the cover camouflage, have similar angle dependence in the spectral response, meaning that their reflective spectra will blueshift simultaneously and that the difference between them will not increase with increasing observation angles (a detailed discussion can be found in Fig. S15). As shown in Fig. 4a, b, although the reflection spectra of the compound nanosieves drifted according to the transitions of the observation angles, the spectral differences caused by perturbations in *D* (Fig. 4a) or *h* (Fig. 4b) had no visible increase.

To experimentally demonstrate the robustness of information hiding, we observed the sample in Fig. 3b, iii (with perturbation 2) under objectives with different numerical apertures (NAs), which provided different maximum illuminating angles (Fig. 4c-f). Consequently, under the objectives of ×50 (NA = 0.8, with a maximum incidence angle of 53.1°) and ×100 (NA = 0.9, with a maximum incidence angle of 64.2°), the background color transformed into green from the original yellow (Fig. 4e, f). Remarkably, even if placed under a ×100 objective (NA = 0.9), the concealed “SYSU” stayed invisible (Fig. 4f), verifying the angle-independent information hiding ability of the perturbative countersurveillance strategy and its stability while working under ambient conditions.

Compared with previous information encryption methods based on photonic structures, our perturbative strategy exhibits three unique functions, which have seldom been considered before. First, an on-demand cover image can be designed to conceal the real classified information. With the help of the cover camouflage information, the hidden message can be stored without being noticed and transmitted without arousing suspicion even when there is a supervising third party. Second, our method possesses the property of countersurveillance, which means it can self-alarm upon leaking of the concealed information. Third, after a dedicated design of the perturbations used for the information encryption, near-perfect information hiding can be achieved. Since the embedded information can be seen neither from optical spectra nor from SEM images without the correct decryption treatments, it can be concluded that what is concealed in our perturbative strategy is not only the hidden private information but also the existence of the hidden information. To the best of our knowledge, most previous photonic information encryption methods can hardly provide cover information to misguide potential enemies^28–31^. Although several reported approaches involved the idea of cover information^32–34^, they did not possess the capabilities of countersurveillance and near-perfect information hiding, as demonstrated by our perturbative strategy. Therefore, it is believed that this perturbation-induced countersurveillance strategy can not only keep private information out of unauthorized scrutiny even if the cover information is transmitted in public channels but also inherently monitor possible attacks, enabling a highly secure paradigm for covert information storage and exchange.

## Discussion

In summary, we presented a perturbation-induced countersurveillance strategy by manipulating the structural and thermal perturbations in compound nanosieves. The perturbations in the compound nanosieves helped to conceal private information, while large structural changes were used to construct innocuous cover camouflage to erase the existence of hidden information. With the delicately selected perturbations, near-perfect information hiding was achieved in the perturbative metaoptics system. Through the design of the perturbations, the function of countersurveillance was then induced, enabling self-indicators of possible information attacks.

Beyond these highly desired functions for information encryption, to meet all possible complex demands in real-world scenarios, it is also possible to develop other functions based on our approach. For example, one can implement a multiple information hiding scheme using our perturbative method by introducing fake private information to misguide enemies, since the real information and fake information can be concealed by different perturbations. Moreover, the whole set of this information can be embedded in commercial products, maximally reducing the risk of information leakage during transportation (see Fig. S16 for a detailed discussion). In addition, more functionalities based on perturbative metadevices may be developed when involving metamaterials or metasurfaces with multi-dimensional modulation regarding optical properties^38^, material phases^39^ or thermal effects^12^. Our findings not only demonstrate the promising potential for using perturbations as a powerful dimension in the construction of functional materials but also provide a perturbative strategy for securing a safer world of information and internet of things.

## Materials and methods

### Numerical simulation

The simulation results were calculated with the finite-difference time-domain (FDTD) method^40^ using a commercially available FDTD simulation software package from Lumerical Solutions. The average value and root-mean-square (r.m.s.) of the aluminum island film thickness were set as 4 nm and 8 nm, respectively. The corr lengths (average correlation lengths of roughness) were set as 10 × 10 nm^2^. The size of the unit cell was set as 300 × 300 nm^2^. The permittivity of aluminum was taken from Palik^41^. The refractive index of the ZEP resist was measured by a SENTECH Spectroscopic Ellipsometer SE 800 PV (Fig. S17).

### Optical characterization

The optical images of the samples were captured using an upright reflective microscope (Olympus BX51M, Olympus Inc.) equipped with a digital camera (DCC1645C, Thorlabs). The narrowband light sources used in this work were obtained by inserting narrowband filters with corresponding wavelengths into the microscope, and all of their bandwidths were 10 nm. The experimental reflection spectra were measured by a spectrograph (SP2500, Princeton Instruments) connected to the microscope, and an objective of ×10 (NA = 0.3) was adopted in the measurements.

### Morphology characterization

The SEM images were taken by a Zeiss Auriga-39–34 (Oberkchen, Germany) microscope operating at 5.0 kV. The relative heights of the samples were measured by a profilometer (Alpha-Step D-600, KLA Tencor).

### Fabrication

The 100-nm aluminum reflecting layer was deposited onto a silicon substrate by an electron beam evaporation system (DE400, DE Technology) with a speed of 2 Å/s, and a layer of electron resist (ZEP) was spin-coated onto the aluminum reflector, followed by a baking process at 180 °C for 10 min. The designed patterns of holes were written in the ZEP resist with a current of 2 nA, a dose of 210, a resolution of 1 nm and a beam step size of 5 nm using an electron beam lithography system (EBPG5000+, Riath). The sample was subsequently developed in dimethyl benzene for 70 s and then immersed in isopropyl alcohol for 30 s to form the designed distribution of unit holes in the ZEP resist. Then, an aluminum plasmonic cap was coated onto the as-fabricated structures under a sputter current of 100 mA with a duration of 20 s in a Quorum Q150T ES sputtering system to form nanohole-based metasurfaces. The thicknesses of the ZEP resist in the demonstrated badge samples were approximately 390 nm. The thermal processes for perturbation 2 were performed at 180 °C for 1 min using a hotplate (KW-4AH-350, Chemat Scientific).

## Supplementary information

Supplementary Information for Perturbative countersurveillance metaoptics with compound nanosieves

## References

[1] Poddubny A (2013). Hyperbolic metamaterials. Nat. Photonics.

[2] Schuller JA (2010). Plasmonics for extreme light concentration and manipulation. Nat. Mater..

[3] Khanikaev AB, Shvets G (2017). Two-dimensional topological photonics. Nat. Photonics.

[4] Xu MS (2013). Graphene-like two-dimensional materials. Chem. Rev..

[5] Kildishev AV, Boltasseva A, Shalaev VM (2013). Planar photonics with metasurfaces. Science.

[6] Zhang S (2008). Plasmon-induced transparency in metamaterials. Phys. Rev. Lett..

[7] Valentine J (2008). Three-dimensional optical metamaterial with a negative refractive index. Nature.

[8] Zhang X, Liu ZW (2008). Superlenses to overcome the diffraction limit. Nat. Mater..

[9] Khorasaninejad M, Capasso F (2017). Metalenses: versatile multifunctional photonic components. Science.

[10] Wang SM (2018). A broadband achromatic metalens in the visible. Nat. Nanotechnol..

[11] Ni XJ (2015). An ultrathin invisibility skin cloak for visible light. Science.

[12] Li Y (2019). Thermal meta-device in analogue of zero-index photonics. Nat. Mater..

[13] Yu NF, Capasso F (2014). Flat optics with designer metasurfaces. Nat. Mater..

[14] Minovich AE (2015). Functional and nonlinear optical metasurfaces. Laser Photonics Rev..

[15] Lin D (2014). Dielectric gradient metasurface optical elements. Science.

[16] Miao ZQ (2015). Widely tunable terahertz phase modulation with gate-controlled graphene metasurfaces. Phys. Rev. X.

[17] Stockman MI (2018). Roadmap on plasmonics. J. Opt..

[18] Vahala KJ (2003). Optical microcavities. Nature.

[19] Baba T (2008). Slow light in photonic crystals. Nat. Photonics.

[20] Bittner S (2018). Suppressing spatiotemporal lasing instabilities with wave-chaotic microcavities. Science.

[21] Ozbay E (2006). Plasmonics: merging photonics and electronics at nanoscale dimensions. Science.

[22] Wei H (2018). Plasmon waveguiding in nanowires. Chem. Rev..

[23] Kuznetsov AI (2016). Optically resonant dielectric nanostructures. Science.

[24] Benz F (2016). Single-molecule optomechanics in “picocavities”. Science.

[25] Zhou ZK (2019). Quantum plasmonics get applied. Prog. Quantum Electron..

[26] Hu CQ (2018). New design for highly durable infrared-reflective coatings. Light. Sci. Appl..

[27] Matlack KH (2018). Designing perturbative metamaterials from discrete models. Nat. Mater..

[28] Li JX (2018). Addressable metasurfaces for dynamic holography and optical information encryption. Sci. Adv..

[29] Dong FL, Chu WG (2019). Multichannel-independent information encoding with optical metasurfaces. Adv. Mater..

[30] Zhao RZ (2018). Multichannel vectorial holographic display and encryption. Light. Sci. Appl..

[31] Yoon G (2018). “Crypto-display” in dual-mode metasurfaces by simultaneous control of phase and spectral responses. ACS Nano.

[32] Hu DJ (2018). Laser-splashed three-dimensional plasmonic nanovolcanoes for steganography in angular anisotropy. ACS Nano.

[33] Liu HL (2019). Tunable resonator-upconverted emission (TRUE) color printing and applications in optical security. Adv. Mater..

[34] Xue JC (2015). Scalable, full-colour and controllable chromotropic plasmonic printing. Nat. Commun..

[35] Lo HK, Curty M, Tamaki K (2014). Secure quantum key distribution. Nat. Photonics.

[36] Scarani V (2009). The security of practical quantum key distribution. Rev. Mod. Phys..

[37] Shor PW, Preskill J (2000). Simple proof of security of the BB84 quantum key distribution protocol. Phys. Rev. Lett..

[38] Zhang L (2016). Advances in full control of electromagnetic waves with metasurfaces. Adv. Optical Mater..

[39] Folland TG (2018). Reconfigurable infrared hyperbolic metasurfaces using phase change materials. Nat. Commun..

[40] Farjadpour A (2006). Improving accuracy by subpixel smoothing in the finite-difference time domain. Opt. Lett..

[41] Palik, E. D. *Handbook of Optical Constants of Solids* (Academic Press, San Diego, 1998).

