# Supplementary material for: Perturbative countersurveillance metaoptics with compound nanosieves
Source: Light Sci Appl. 2019 Nov 15;8:101. doi: 10.1038/s41377-019-0212-4 (PMC6858309; doi:10.1038/s41377-019-0212-4)
Supplement: Supplementary file 1 — Supplementary Information for Perturbative countersurveillance metaoptics with compound nanosieves [file 41377_2019_212_MOESM1_ESM.docx]

**Supplementary Information for “Perturbative countersurveillance metaoptics with compound nanosieves”**

Jiancai Xue, Zhang-Kai Zhou^*^, Limin Lin, Chao Guo, Shang Sun, Dangyuan Lei,

Cheng-Wei Qiu^*^, Xue-Hua Wang^*^

**Fig. S1** **Colours of compound nanosieves with rough plasmonic caps............................................ 2**

**Fig. S2 Spectral properties of** **the** **compound nanosieves regarding *h* and *D*................................ 3**

**Fig. S3 Spectral properties of the compound nanosieves regarding Δ*h*......................................... 4**

**Fig. S4 A scheme of information hiding in the perturbative metaoptics........................................ 5**

**Fig. S5 The reflection spectra corresponding to the sample in Fig. 1d........................................... 7**

**Fig. S6 Information hiding under narrowband light source with wavelengths away from reflective valley..............****................................................................................. 8**

**Fig. S7 Spectral sensitivity regarding the diameters of nanosieves and the changes of the diameters..................................................................................................... 9**

**Fig. S8 C****ompound nanosieves for information expressing with ultrahigh resolution................** **10**

**Fig. S9 Reflection spectra of compound nanosieves with the same porosity…………………... 11**

**Fig. S10 Morphological invisibility of compound nanosieves with small perturbation in shapes............................................................................................................ 12**

**Fig. S11 Thermal effect of resist with holes (the dielectric spacer)............................................... 13**

**Fig. S12 No trace of the concealed message before the thermal process...................................... 14**

**Fig. S1****3 Spectral shift of the compound nanosieves before and after the thermal process….... 15**

**Fig. S14 Morphological invisibility of the hidden message after adding perturbation 2…........ 16**

**Fig. S15 Relationships between reflective peak wavelength (λ*_p_*) (a), peak wavelength difference (∆λ*_p_*) (b) and refractive angle (θ) in a thin film interference model..... 17**

**Fig. S16 A multiple scheme of information hiding in the perturbative countersurveillance metaoptics................................................................................................... 19**

**Fig. S17 Measured refractive index of ZEP resist.......................................................................... 20**

**
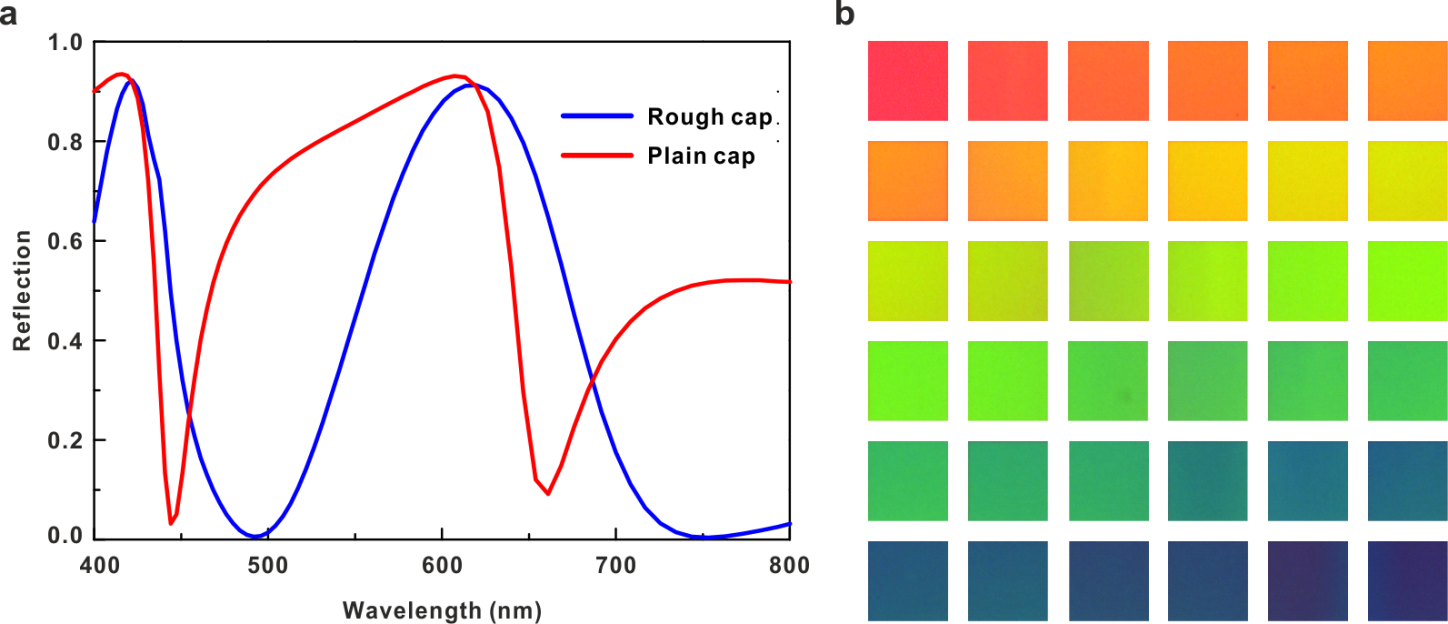
**

**Figure S1 Colours of compound nanosieves with rough plasmonic caps.** (**a**) The simulated reflection spectra of compound nanosieves with rough cap (the blue line) and with plain cap (the red line). The thickness of the dielectric cap is 385 nm, the average thickness of the rough Al cap 4 nm, the thickness of the plain Al cap 5 nm. The rough plasmonic cap creates narrower reflective peaks, making it more suitable to generate colours with high saturations and wide colour ranges. (**b**) Experimentally generated colours ranging from red to green to blue using compound nanosieves with diameters for 0 to 260 nm.


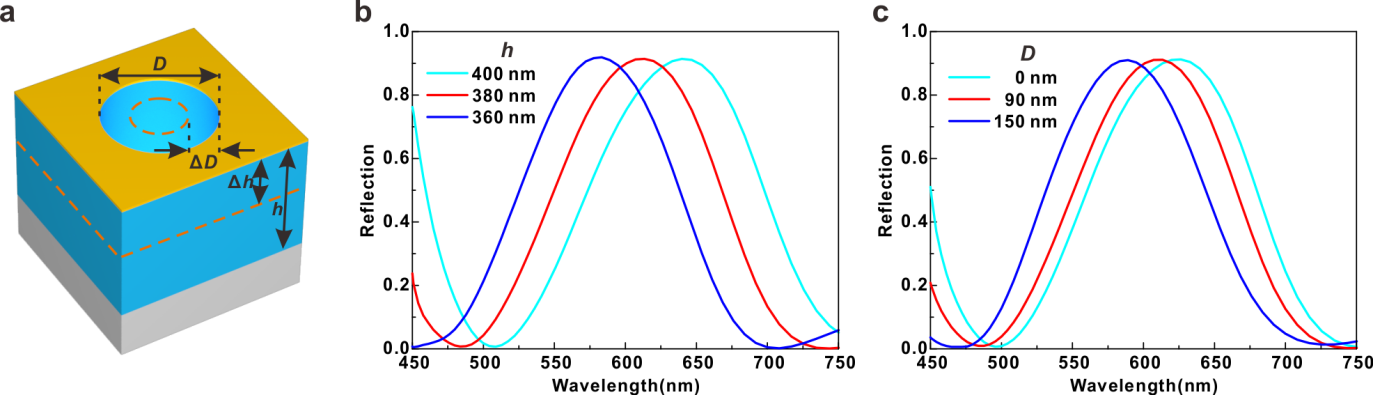


**Figure S2** **Spectral properties of the compound nanosieves regarding *h* and *D*.** (**a**) The model of the compound nanosieves. The formation of the final spectra of the compound nanosieve stems from the combination of plasmonic resonances of the metallic cap and the thin film interference. Therefore, *h* and *D* are key parameters in the modulation of the reflection spectra because they determine the optical paths in the dielectric spacer. (**b**) The relationship between the reflection spectra of the compound nanosieves and the thickness of the dielectric spacer (*h*). The corresponding reflective peaks red shift as *h* increases. (**c**) The relationship between the reflection spectra of the compound nanosieves and the diameters of the nanoholes (*D*). The corresponding reflective peaks blue shift as *D* increases.


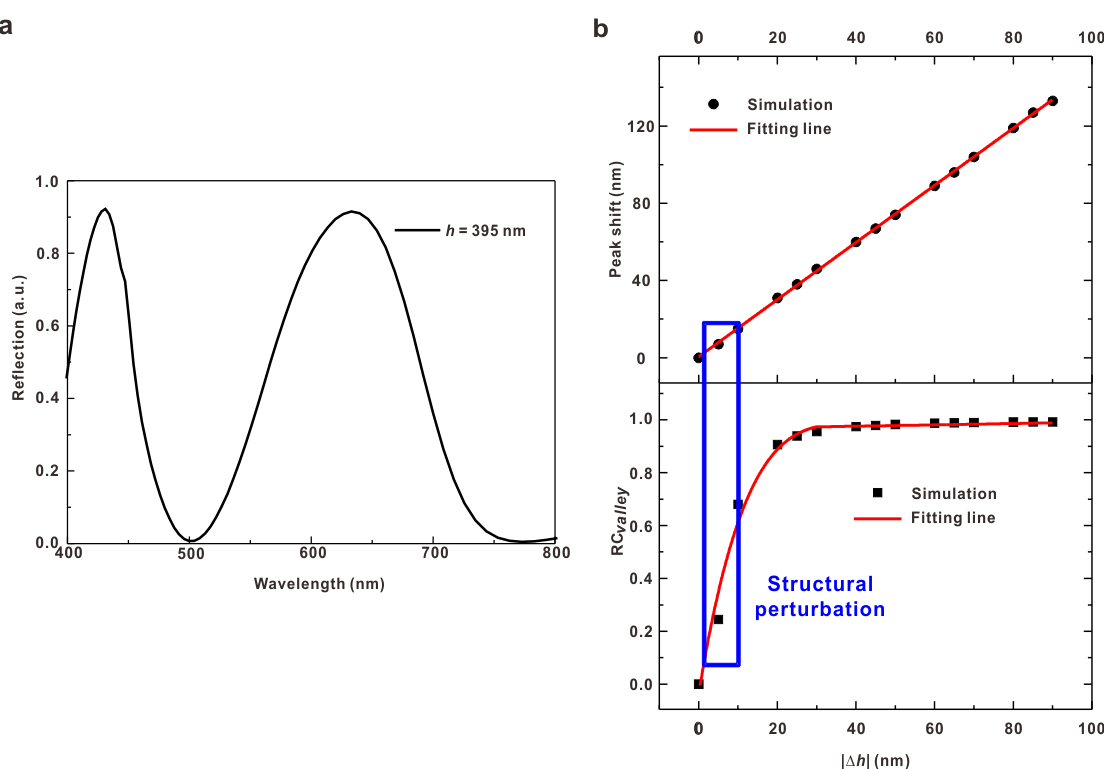


**Figure S3** **Spectral properties of the compound nanosieves regarding Δ*h*.** (**a**) The simulated reflection spectra of the compound nanosieves with *h* = 395 nm and *D* = 0 nm. One reflective valley is located at about 500 nm. (**b**) The relationships between peak shifts or *RC_valley_* (reflection contrast at the original valley wavelength, 500 nm) and Δ*h* with the given height *h* = 395 nm (*D* = 0 nm). In this simulation, *h* was changed from 395 nm to 305 nm. Similar to the case of Δ*D*, *RC_valley_* increases rapidly as Δ*h* increases in the structural perturbation region (marked by blue square), while the corresponding peak shifts remain small.


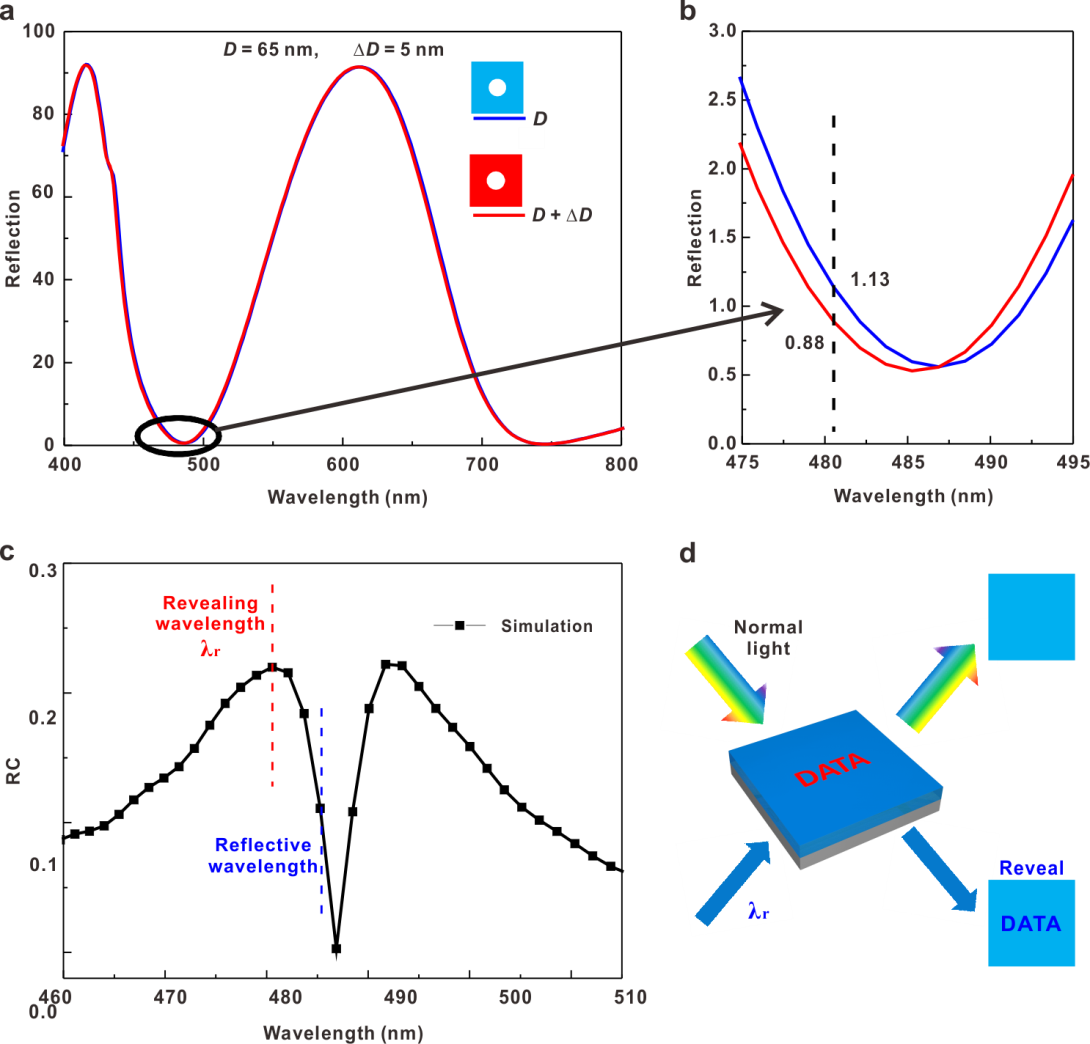


**Figure S4** **A scheme of information hiding in the perturbative metaoptics****.** (**a**) Simulated reflectance spectra of two compound nanosieves with a slight structural perturbation (*D* = 65 nm, Δ*D* = 5 nm). The dielectric layer was set as ZEP resist of 385 nm, and the corresponding diameters of holes were 65 nm (blue curve) and 70 nm (red curve). These two spectra are difficult to differentiate because of their small spectral offset of only about 2 nm. Such a small spectral offset can be used to well conceal data. (**b**) The magnified reflection spectra in the labeled circle in **a**. There is a *RC* = 0.22 (0.88% and 1.13%) at a wavelength near the reflective valley (labeled by the dashed line), significant enough to be differentiated by human eyes. (**c**) RC spectra around the reflective valley wavelength. This figure is calculated based on the simulated spectra in **a**, taking the case of *D* = 70 nm as the feature of interest. Here, the wavelength marked in **b** is spotted by the red dashed line as the revealing wavelength *λ_r­­_* (denoted as *λ_d­­_* in the main text), and the position of reflective valley is marked by the blue dashed line. The maximum *RC* does not appear at the reflective valley but at specific wavelengths near the valley. The existence of a zero point is resulted from the cross point of the spectra of these two compound nanosieves with structural perturbation. (**d**) Schematic illustration of information hiding enabled by the perturbative compound nanosieves, where slight perturbations are used to hide information. The hidden message (‘DATA’) is invisible under white light illumination (upper case) but distinguishable when illuminated by light with the revealing wavelength (*λ_r_*) (lower case)


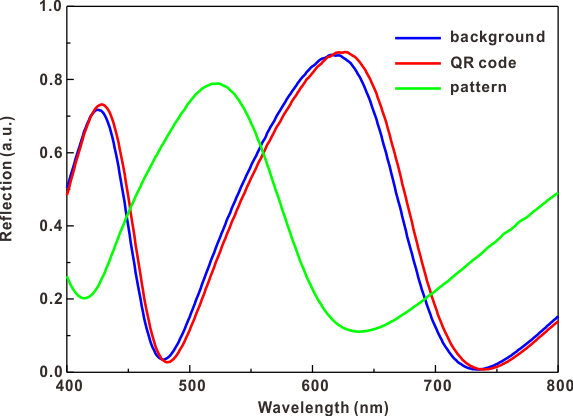


**Figure S5** **The reflection spectra corresponding to the sample in Fig. 1d.** The blue curve and the red curve correspond to the background and the concealed QR code, respectively, showing a small spectral offset. The green curve corresponds to the patterns in the badge.

**
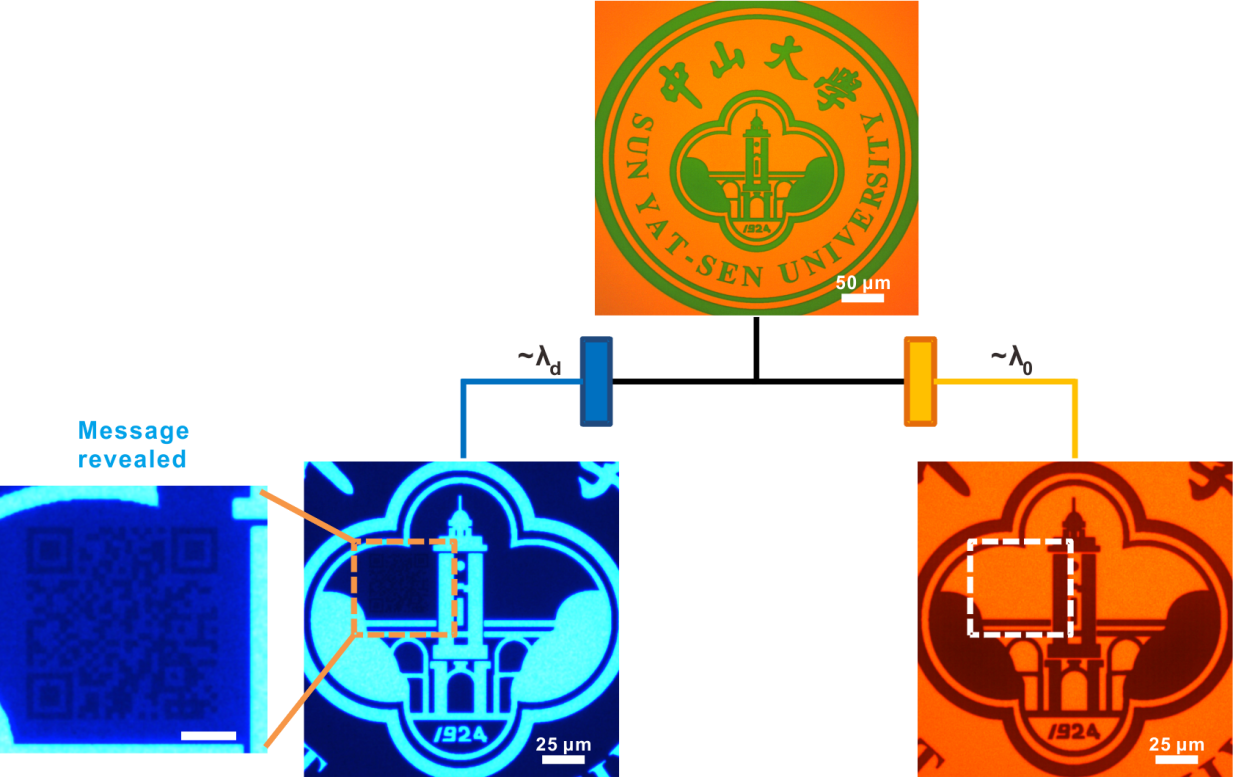
**

**Figure S6** **Information hiding under narrowband light source with wavelengths away from reflective valley.** While the concealed message (the QR code) can be revealed by filter with wavelengths near the design wavelength (*λ_d_* = 480 nm, the lower-left case), this information would be well hidden when the applied filters just enables ordinary wavelengths away from the reflective valley (*λ_o_* = 600 nm as an example, the lower-right case). The scale bar in the magnified image of the QR code is 10 μm.


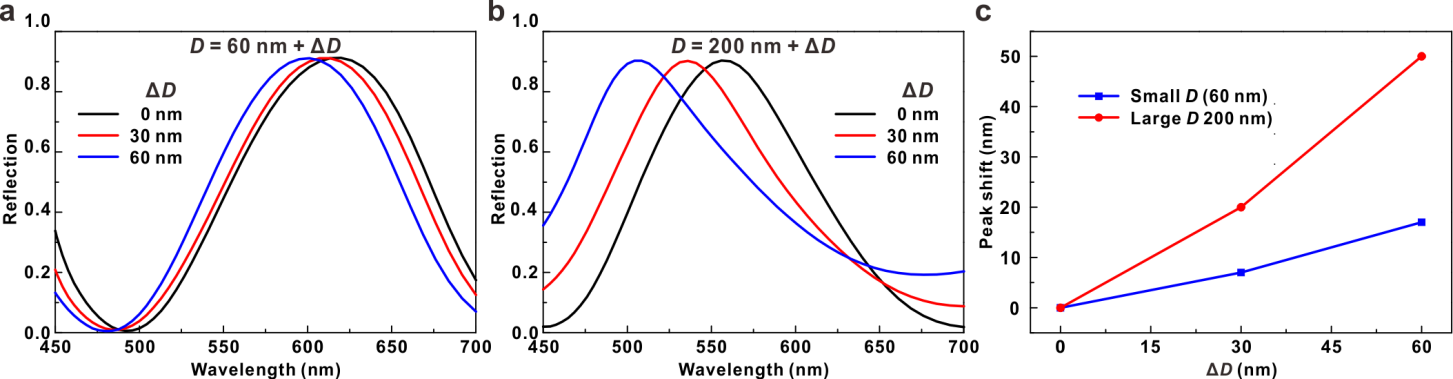


**Figure S7 Spectral sensitivity regarding the diameters of nanosieves and the changes of the diameters. (a)** The case of a small base diameter (*D* = 60 nm). (**b**) The case of a much larger base diameter (*D* = 200 nm). (**c**) Shifts of reflective peak wavelengths different *D* against the changes of diameters (∆*D*). Compared with the case of smaller *D* (blue line), the spectra of larger *D* have larger peak shifts when given the same ∆*D* (red line), showing much higher spectral sensitivity to the diameter changes.

**
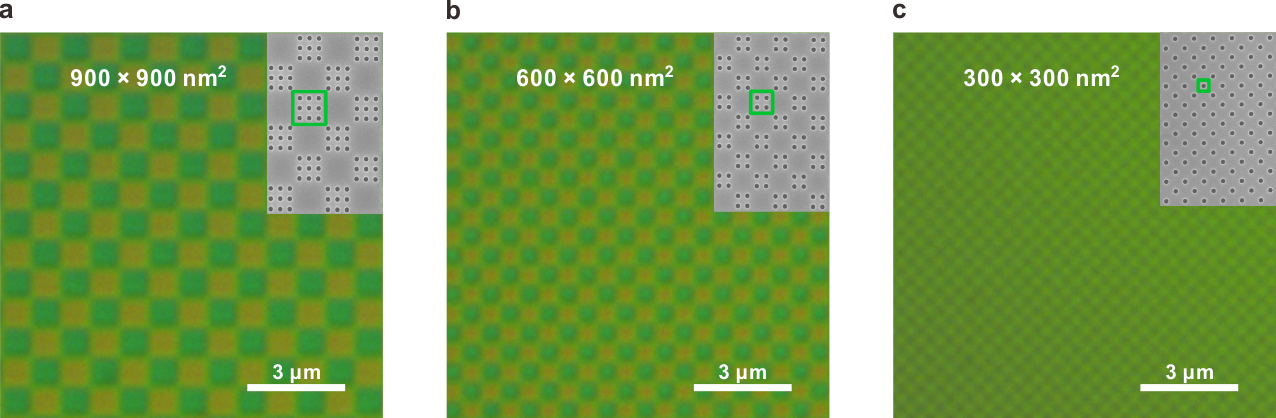
**

**Figure S8** **Compound nanosieves for information expressing with ultrahigh resolution.** (**a-c**) Microscope pictures of ultra-small pixels of the perturbative compound nanosieves with size of 900 × 900 nm^2^ (**a**), 600 × 600 nm^2^ (**b**), and 300 × 300 nm^2^ (**c**). Inserted images at the upper-right corners are the corresponding SEM morphology with pixel units labeled by green squares. It can be seen that the minimum pixel size is the same as the period of the structural unit cells which 300 nm, bringing about an ultrahigh resolution of near 100,000 dots per inch (d.p.i.).


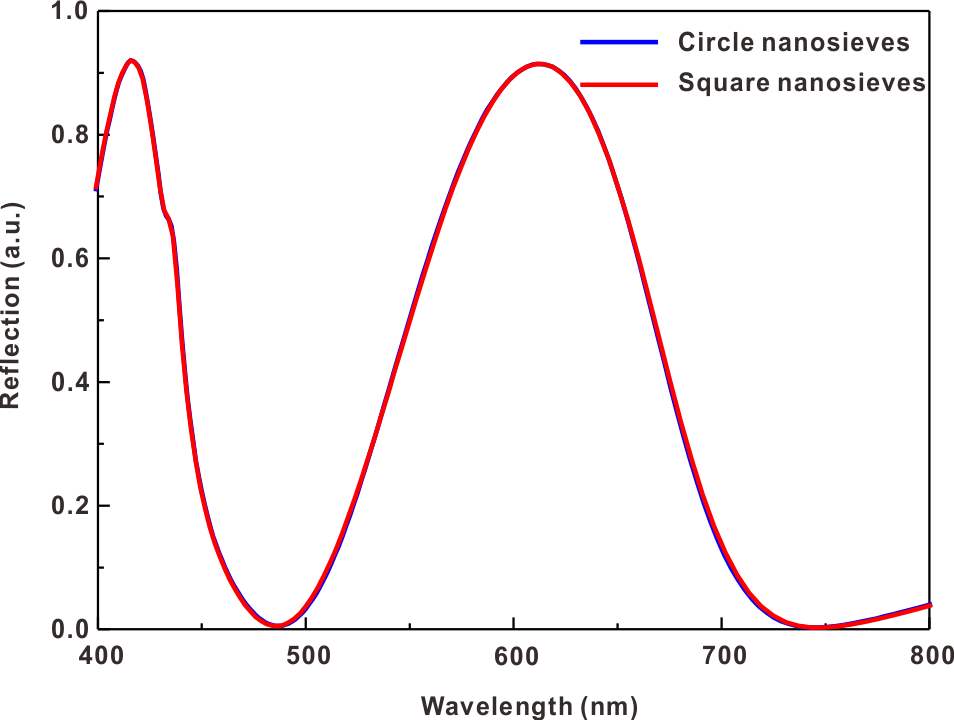


**Figure S9** **Reflection spectra of compound nanosieves with the same porosity.** The simulated reflection spectra of the compound nanosieves with circular holes (*D* = 65 nm) and with square holes (*L* = 58 nm) is represented by the blue curve and the red curve, respectively. Both porosities of these two structures are *P* = 3.7%. These two spectra overlap well with each other, indicating that these two structures cannot be distinguished spectrally.


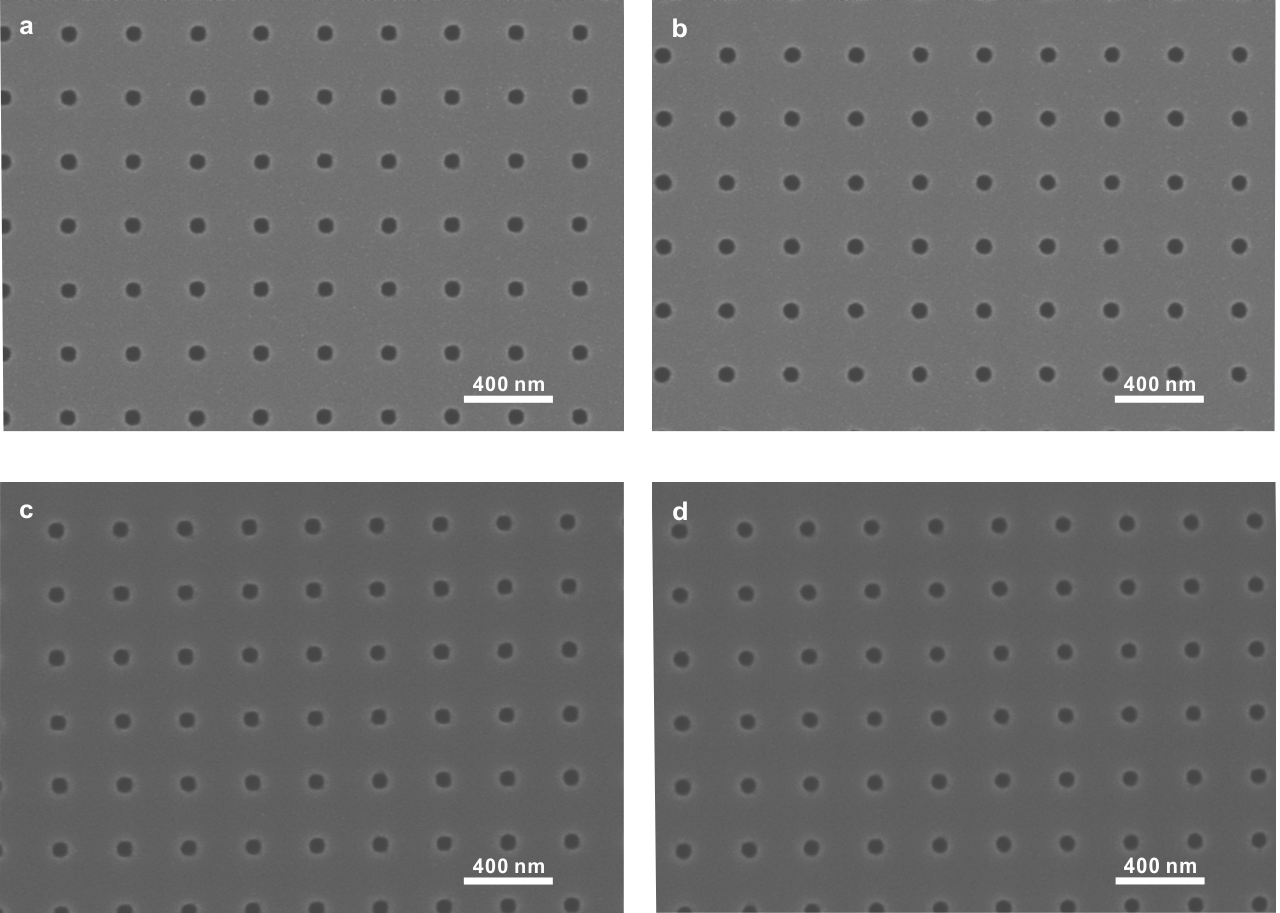


**Figure S10 Morphological invisibility of compound nanosieves with small perturbation in shapes.** (**a**) Rounded-square compound nanosieves. (**b**) Circular compound nanosieves. (**c**) Rounded-square compound nanosieves after the thermal process. (**d**) Circular compound nanosieves after the thermal process. Since the rounded square holes had round corners, their size (*L* = 61 nm) was slightly larger than that of the simulated square holes (*L* = 58 nm, Fig. S7).


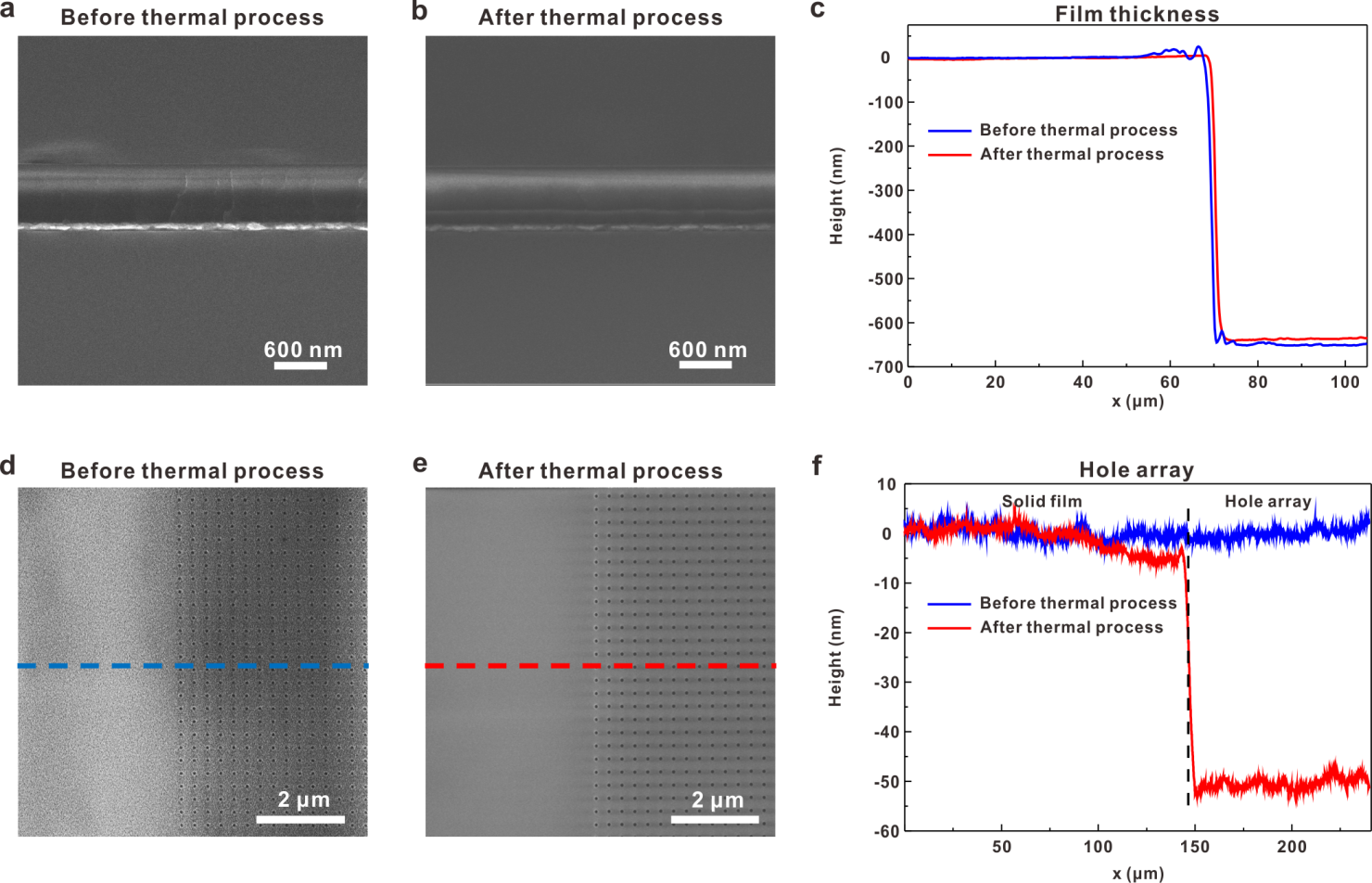


**Figure S11 Thermal effect of resist with holes (the dielectric spacer).** (**a-b**) The SEM images of the cross-sections of an ARP6200 resist on a metal-on-silicon substrate before (**a**) and after (**b**) a thermal process at 180°C for 1 min. (**c**) The thicknesses of the resist layer before (the blue curve) and after (the red curve) the thermal process measured by a profilometer. The thicknesses of the resist reduced slightly from 650 nm to 640 nm, with a decrease of 10 nm. (**d-e**) The SEM images of a hole array (*D* = 65 nm, *P* = 300 nm) on the resist before (**d**) and after (**e**) the thermal process. (**f**) The relative height of hole array regarding the resist before (the blue curve) and after (the red curve) the thermal process measured by the profilometer, corresponding to the regions marked by blue line in **d** and red line in **e**, respectively. Although there was no step of height before baking, a distinct step of approximate 50 nm appeared between the flat resist (small SV ration) and the hole array (large SV ratio) after the thermal process, meaning that the resist with holes shrank more than the flat resist region during the thermal process. This result shows that the thickness of a resist layer with larger SV ratio can decrease more than that with smaller SV ratio when a thermal process is applied.

**
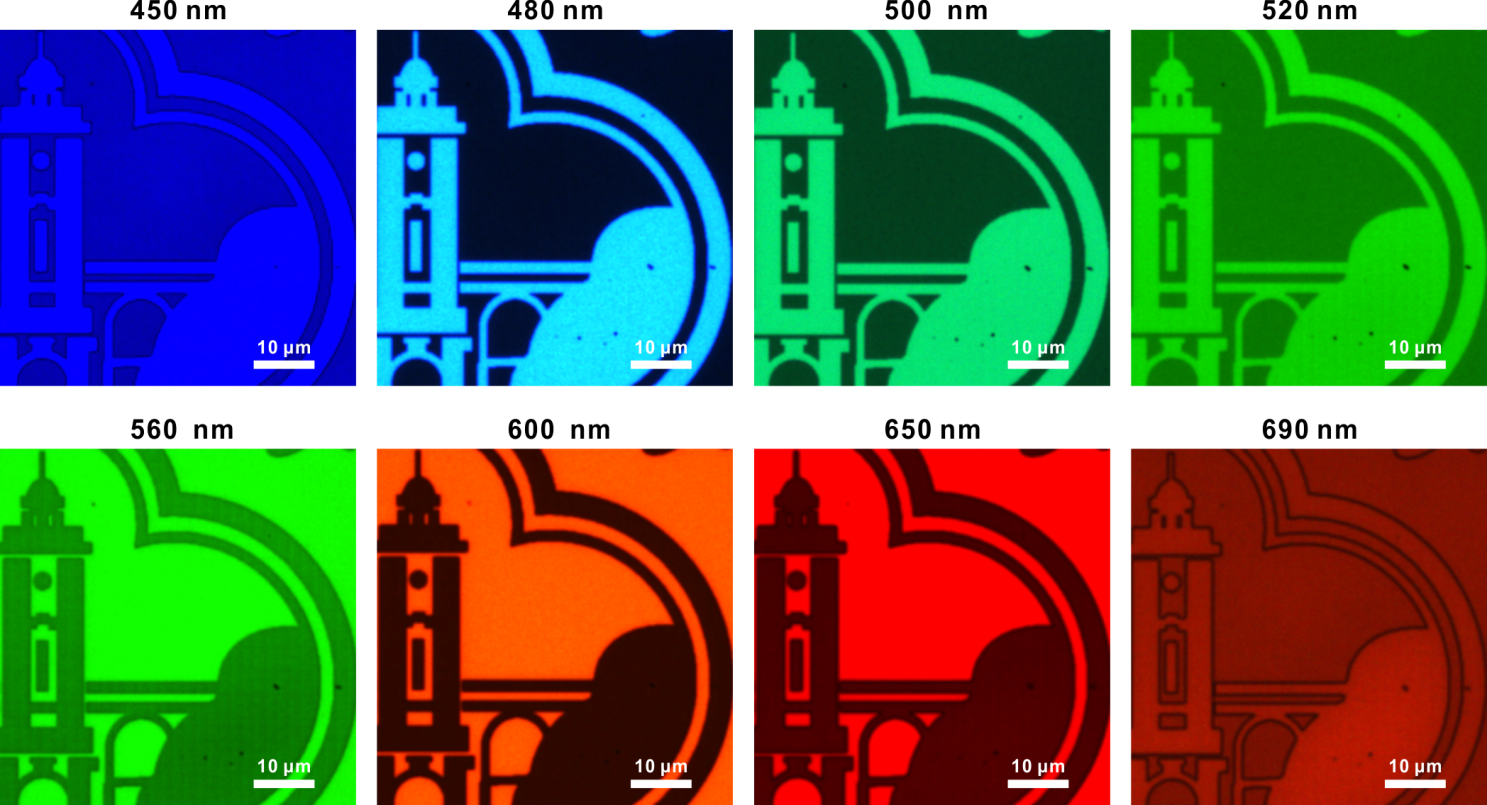
**

**Figure S12** **No trace of the concealed message before the thermal process.** These microscope pictures show the images in the perturbative countersurveillance metaoptics containing hidden information (corresponding to the sample in Fig. 3b, iii), with wavelengths of the applied narrowband light source ranging from 450 nm to 690 nm, showing no trace of the concealed message (‘SYSU’).


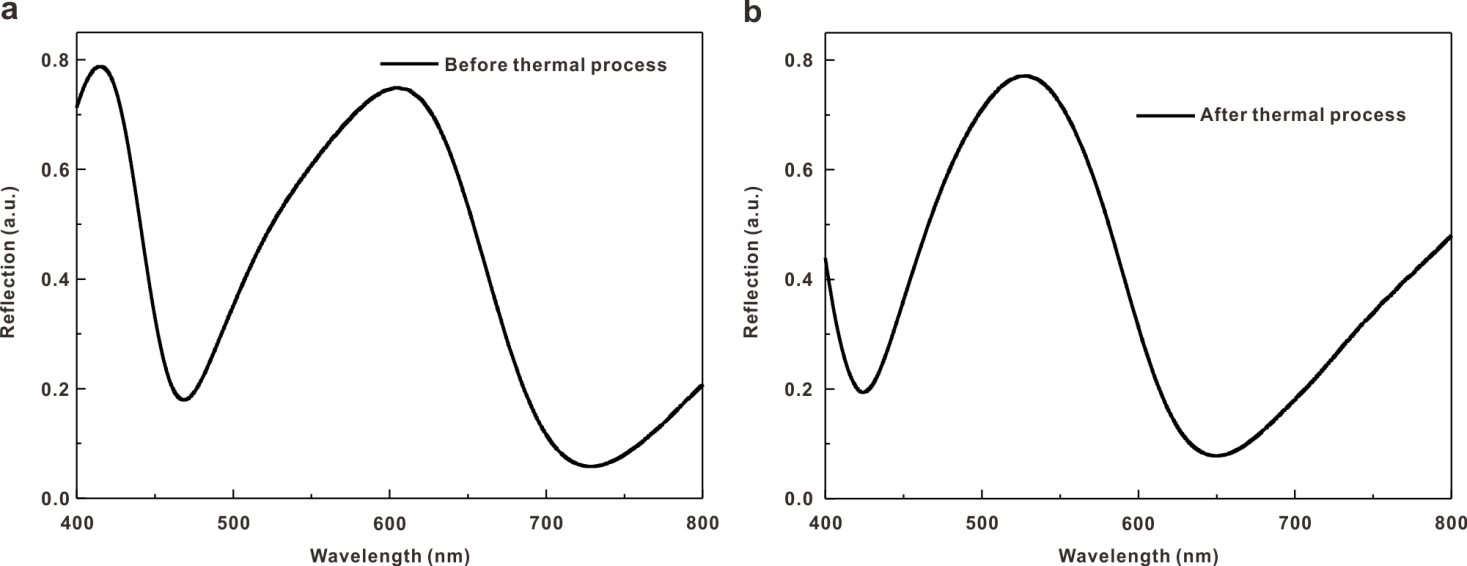


**Figure S13 Spectral shift of the compound nanosieves before and after the thermal process.** (**a-b**) The reflection spectra of the background areas before (**a**) and after (**b**) the thermal process, with the longer reflective valleys shifted from 727 nm to 644 nm. The sample is corresponding to that in Fig. 3b.


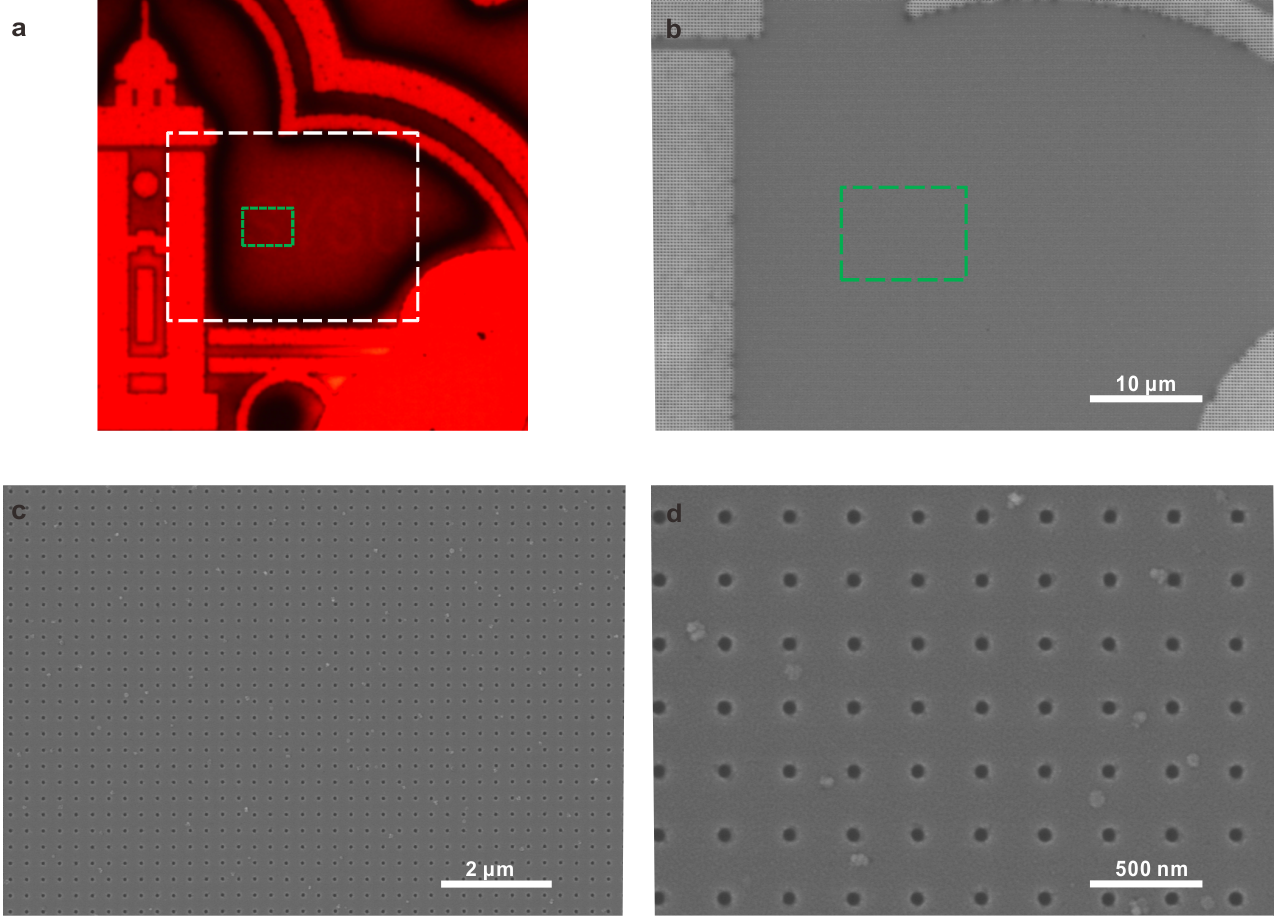


**Figure S14** **Morphological invisibility of the hidden message after adding perturbation 2.** (**a**) The microscope picture of the sample in Fig. 3, v with the revealed ‘SYSU’. (**b**) The SEM image of the region marked by white square in **a**. (**c**) The magnified SEM image around the region marked by green squares in **a** and **b**. There is no sign of the concealed message. (**d**) A further magnified SEM image. It is very difficult to find the embedded pattern under SEM because of the high similarity between the rounded square holes of the ‘SYSU’ and the circular hole of the background used in the perturbative countersurveillance metaoptics.

**
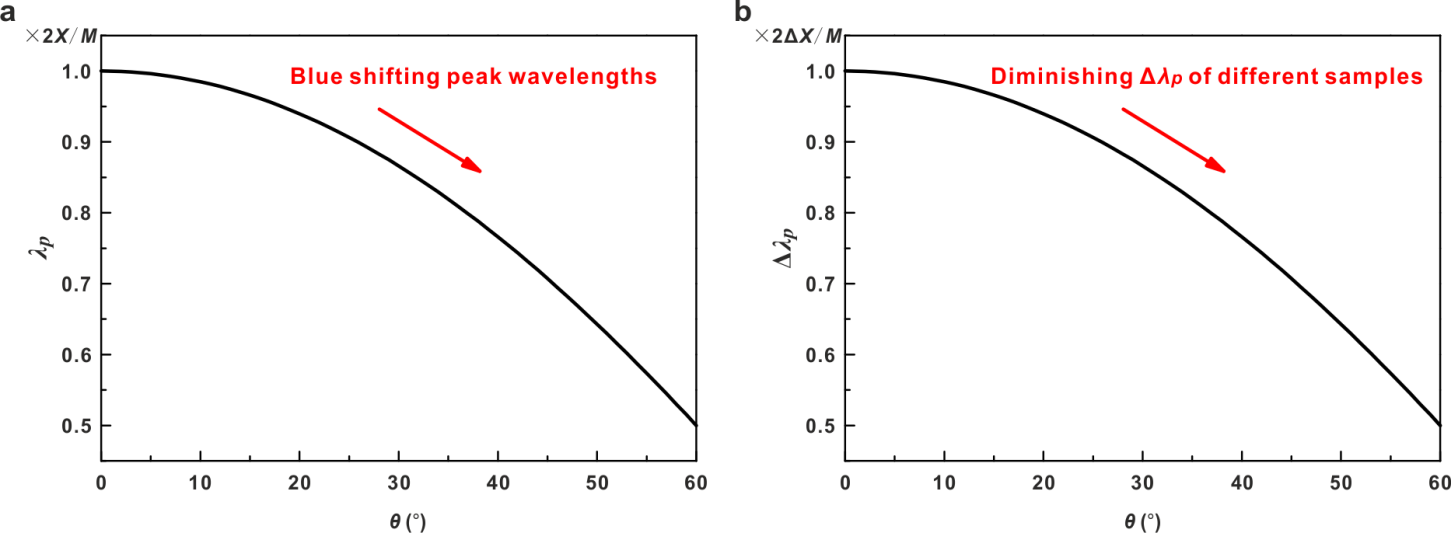
**

**Figure S15 Relationships between reflective peak wavelength (*λ_p_*) (a), peak wavelength difference (∆*λ_p_*) (b) and refractive angle (*θ*) in a thin film interference model, all showing a monotonic decreasing tendency against *θ*.** The shift of reflective peak wavelengths (***λ_p_***) can be understand by a thin film interference model which is an origin of the reflective peaks. In the thin film interference model, we have (m - 0.5)*λ_p_* = 2*n_eff_d*cos*θ*, where m is an integer larger than zero representing interference order, *λ_p_* the peak wavelengths, *n_eff_* the effective refractive index of the thin dielectric spacer, *d* the thickness of the spacer, and *θ* the refractive angle (*Handbook of Optical Constants of Solids.* Ch 7. Academic, 1998; *Nano Lett.* **14,** 3374, 2015). Here we replace (m – 0.5) by M (= m – 0.5), and define a structural parameter *X* = *n_eff_d*, the value of which is determined by the structures. Then, we get *λ_p_* = (2/M)*X*cos*θ*. Since the spectral shapes of the compound nanosieves have no substantial change regarding different incident angles (see Fig. 4a,b), the peak wavelength *λ_p_* is a predominant parameter characterizing the optical property of the structure, and thus the peak wavelength difference (∆*λ_p_*) between different nanosieves can be an important factor representing the difference of their optical responses. As for angle-dependence of optical response, all compound nanosieves have the same form determined by the above equation despite of their specific structural parameters (denoted by *X*), which means that their optical responses always change with the same tendency regarding the incident angles (peak wavelengths decrease/blue-shift corresponding to increased incident angle, Fig. S15a). In terms of the spectral difference between different nanosieves, one can name two different samples by Sample 1 and Sample 2, and get *λ_p1_* = (2/M)*X*_1_cos*θ* and *λ_p2_* = (2/M)*X*_2_cos*θ*. Thus the parameter determines their spectral difference will be ∆*λ_p_* = (2/M)∆*X*cos*θ*, where ∆*X* = *X*_2_-*X*_1_. ∆*X* will be fixed with two determined samples, then their largest ∆*λ_p_* equals to (2/M)∆*X* in the case of normal incidence (*θ* = 0), and ∆*λ_p_* decreases as *θ* increases (Fig. S15b). This result means that when the observing angle increases, the spectral difference between two nanosieves structures with and without perturbation would be diminished instead of being enlarged, indicating the possibility of higher security for information hiding.

**
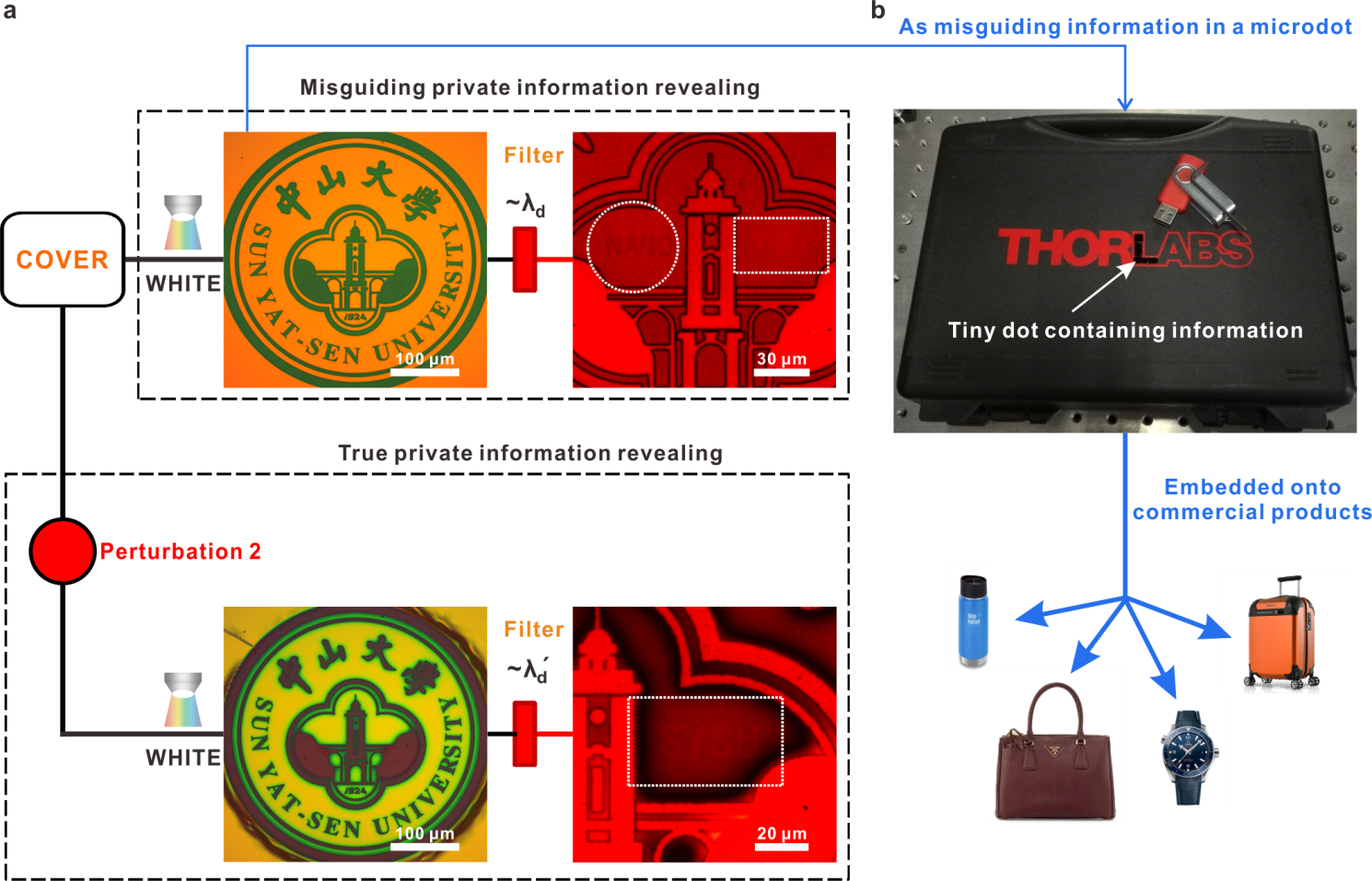
**

**Figure S16 A multiple scheme of information hiding in the perturbative countersurveillance metaoptics.** (**a**) Together with real private information (‘SYSU’), fake private information (‘NANO’) can be embedded to misguide the eavesdropper when they perform information attacks. The misguiding message (‘NANO’) was concealed by the elementary information hiding scheme just based on size perturbation (Δ*D* = 5 nm), which is corresponding to the method shown in Fig. 1d. (**b**) The cover camouflage (the background badge) can further work as misguiding information in the form of microdot (a classical form of steganography), consisting of a multiple scheme of information hiding with camouflages, and the whole panel can be embedded onto commercial products for safely transferring.


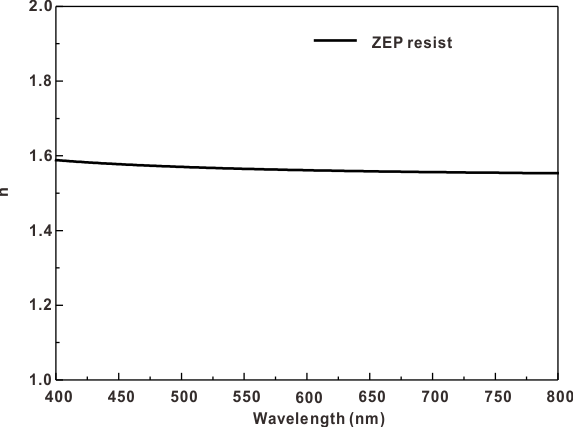


**Figure S17 Measured refractive index of ZEP resist.** The refractive index of ZEP resist was measured by SENTECH Spectroscopic Ellipsometer SE 800 PV.
